# Complete Genome Sequence of Geobacter sulfurreducens Strain YM18, Isolated from River Sediment in Japan

**DOI:** 10.1128/genomeA.00352-18

**Published:** 2018-05-10

**Authors:** Kengo Inoue, Yoshitoshi Ogura, Yoshihiro Kawano, Tetsuya Hayashi

**Affiliations:** a Department of Biochemistry and Applied Biosciences, Faculty of Agriculture, University of Miyazaki, Miyazaki, Japan; b Department of Bacteriology, Faculty of Medicine Sciences, Kyushu University, Fukuoka, Japan

## Abstract

Geobacter sulfurreducens is known to be a dominant species in the anode biofilms of microbial fuel cells. Here, we report the complete genome sequence of G. sulfurreducens strain YM18. Strain YM18 was isolated from a biofilm formed on an anode poised at −400 mV (versus an Ag/AgCl electrode) in a bioelectrochemical system.

## GENOME ANNOUNCEMENT

Geobacter sulfurreducens and its close relatives are known to be dominant species in the biofilms formed on anodes in microbial fuel cells (1). Their extracellular electron transfer ability, by direct contact and electrically conductive nanowires, enables them to produce higher current densities than any other microorganism. The complete genome sequence of G. sulfurreducens strain PCA (the type strain of this species) was previously determined (2), revealing that the strain has many two-component regulators and *c*-type cytochromes. The genome of strain KN400, which can produce much higher current densities than strain PCA, was also completely sequenced and compared with that of strain PCA (3, 4). Here, we report the complete genome sequence of G. sulfurreducens strain YM18. Its original inoculum was collected from river sediment from the Yae River in Miyazaki, Japan. Electricity-generating microorganisms were enriched in a bioelectrochemical system in which acetate and an anode poised at −400 mV (versus an Ag/AgCl electrode) were the sole electron donor and acceptor, respectively. Strain YM18 was isolated from the biofilm and it had a significantly higher electricity-producing ability than strain PCA.

Strain YM18 was anaerobically grown at 30°C on a medium in which acetate and fumarate are the sole electron donor and acceptor, respectively, as previously described (5). Genomic DNA was purified with the genomic-tip 100/G column and genomic DNA buffer set (Qiagen) according to the manufacturer’s instructions. Sequencing was performed on the 454 GS FLX Titanium (Roche) and MiSeq (Illumina) platforms. A total of 86,141 8-kb paired-end reads from the 454 GS FLX Titanium and 1,164,157 151-bp fragment reads from the MiSeq were assembled by Newbler version 2.9 (Roche), yielding 26 contigs (>500 bp) and a single scaffold. All gaps were closed by sequencing of Gap-spanning PCR products using an ABI 3730 DNA analyzer (Applied Biosystems). Genome annotation was performed using Prokka (6).

The genome of strain YM18 consists of a 3,726,411-bp chromosome with a G+C content of 60.7%. No plasmids were found. The chromosome contains 2 rRNA operons, 49 tRNA genes, and 3,327 protein-coding sequences (CDSs). The genome size and the number of CDSs were similar to those of KN400 (3,714,272 bp and 3,389 CDSs) but less than those of PCA (3,814,128 bp and 3,711 CDSs). Compared to the two previously sequenced G. sulfurreducens strains, YM18 contains 220 unique CDSs. G. sulfurreducens strains generally have over 100 *c-*type cytochromes. A slightly smaller number of genes were predicted to encode *c*-type cytochromes in YM18 (101 CDSs) than in PCA and KN400 (106 and 107, respectively). The complete genome sequence of YM18 and more detailed genome comparison with strains PCA and KN400 will identify commonly preserved genetic features for effective electron transfer to electrodes.

### Accession number(s).

The complete genome data have been deposited in DDBJ/GenBank under the accession number AP017912.
